# The Trend of Tuberculosis Case Notification Rates from 1995 to 2022 by Country Income and World Health Organization Region

**DOI:** 10.3390/tropicalmed9120294

**Published:** 2024-12-02

**Authors:** Kobto G. Koura, Anthony D. Harries

**Affiliations:** 1 International Union Against Tuberculosis and Lung Disease, 75001 Paris, France; adharries@theunion.org; 2 MERIT Research Unit (UMR261 MERIT), University of Paris Cité (UPCité), Research Institute for Development (IRD), 75006 Paris, France; 3 Department of Clinical Research, Faculty of Infectious and Tropical Diseases, London School of Hygiene and Tropical Medicine, Keppel Street, London WC1E 7HT, UK

**Keywords:** tuberculosis, case notification rate, low-income countries, lower middle income countries, upper-middle-income countries, higher-income countries, WHO region

## Abstract

Over the past 27 years, three major global TB control strategies have been implemented, and it is important at this stage to evaluate their impact on tuberculosis (TB) case notification rates (CNRs). This study, therefore, analyzed TB CNR trends from 1995 to 2022 across 208 countries and islands, using data from the WHO Global TB Programme database. Countries were classified by income level and population size based on World Bank criteria. The analysis revealed significant disparities in TB CNRs across income groups: Low-income, lower-middle-income, and upper-middle-income countries consistently reported higher CNRs compared to high-income countries. Regional analysis further demonstrated notable variations influenced by both economic and geographical factors. These findings reaffirm the strong link between TB and poverty, underscoring the need for a holistic approach to combat the disease. Efforts must extend beyond enhancing health care access and delivery to addressing the social determinants that drive TB transmission and progression.

## 1. Introduction

Tuberculosis (TB) remains one of the deadliest infectious diseases in the world. In 2019, Cohen et al. estimated that globally, nearly 2 billion people were infected with latent TB [1]. According to the World Health Organization (WHO), approximately 10 million people each year develop active TB [2,3,4,5]. The total estimated number of deaths from TB, including those co-infected with the human immunodeficiency virus (HIV), increased between 2018 and 2021 from 1.4 million to 1.6 million, making TB the world’s second leading cause of death from a single infectious agent after COVID-19.

The first global strategy for TB control was elaborated by WHO early in the 1990s [6]. It was based on the International Union Against Tuberculosis and Lung Disease’s (The Union) model, developed by Dr. Karel Styblo and colleagues [7,8]. For many years, it remained at the core of national TB strategies, particularly in low- and middle-income countries with a high burden of TB. The second global strategy, ‘The Stop TB Strategy’, which built on and enhanced the first strategy to meet the TB-related Millennium Development Goals and the Stop TB Partnership targets, was launched in 2006 [9]. It was successful in halting and reversing the global increase in the incidence of TB. In 2015, WHO launched ‘The End TB Strategy’, which was aligned with the United Nations (UN) Sustainable Development Goals (SDGs) [10]. The overarching goal of this strategy is “A world free of tuberculosis–zero deaths, disease and suffering due to tuberculosis”.

Despite the bold commitments made in the UN’s SDGs, The WHO End TB Strategy, The Stop TB Partnership’s ‘Global Plan’, and the UN High-Level Meeting targets, all of which aim to end TB by 2030 or 2035, there is general agreement that the world is not on track to achieve this goal. While the COVID-19 pandemic made matters worse, it was clear that the deviation from the required trajectory pre-dated COVID-19. However, rather than abandoning these goals, it is important to reassess and reinforce the strategies that have been used in the past and are currently in place. As part of this global effort, we seek to analyze available data to better understand trends in TB case notifications and identify areas where improvements can be made.

The WHO Global Tuberculosis Report provides data each year on TB case notifications by country and WHO region. To our knowledge, no studies have described the TB case notification rate (CNR) by country income at the global level and by WHO region. To contribute to the various TB control strategies, the current paper aims to analyze the trend of TB CNRs between 1995 and 2022 at the global level and in the six WHO regions by income status.

## 2. Materials and Methods

### 2.1. Study Design, Setting, and Data Collection

The study design is a retrospective cohort analysis of TB CNR over a 27-year period, stratified by country income levels and WHO regions.

We selected the countries included in the most recent 2023 WHO Global Tuberculosis Report [11]. TB case notifications reported from 1995 to 2022 for 217 countries and islands by WHO were collected [12]. We collected the World Bank countries’ income classifications [13]. The World Bank data help desk included 218 countries and islands.

The two data sources were merged and compared, and the following countries were excluded.

- In the WHO database, nine countries were excluded as their income from the World Bank database was not available: (1) Anguilla, (2) Netherlands Antilles, (3) Cook Islands, (4) Montserrat, (5) Niue, (6) Serbia & Montenegro, (7) Tokelau, (8) The Bolivarian Republic of Venezuela, and (9) Wallis and Futuna;
- In the World Bank database, nine countries and islands were excluded as TB case notifications were not available from the WHO Report: (1) Channel Islands, (2) Faroe Islands, (3) Gibraltar, (4) Isle of Man, (5) Liechtenstein, (6) St. Martin (French part), (7) Taiwan, China; (8) Virgin Islands (U.S.), and (9) Kosovo.

We, therefore, focused our analysis on 208 countries.

### 2.2. Study Definitions

According to WHO, a TB case notification refers to a person diagnosed with TB and officially reported as a case to national authorities. Data on notified TB cases are systematically collected at the national level and then reported to WHO on an annual basis, the reporting being based on standard case definitions and associated guidance on the recording and reporting of data provided by WHO [11].

The TB case notification rate (CNR) is calculated as follows: CNR = (Numerator/Denominator) × 100,000. Where: The numerator refers to the number of new and relapse TB cases and cases with unknown previous TB treatment history (all forms) notified during the reporting period. The denominator refers to the number of persons (estimated population) in the same reporting period. The World Bank database on population was used [14].

The World Bank assigns the world’s economies to four income groups: low, lower-middle, upper-middle, and high income. The classifications are updated each year on July 1 and are based on the Gross National Income (GNI) per capita of the previous year. GNI measures are expressed in United States dollars (USD) and are determined using conversion factors derived according to the World Bank’s Atlas method [15]. For the current 2024 fiscal year, low-income economies are defined as those with a GNI per capita of USD 1135 or less in 2022; lower middle-income economies are those with a GNI per capita between USD 1136 and USD 4465 in 2022; upper-middle-income economies are those with a GNI per capita between USD 4466 and USD 13,845 in 2022; and high-income economies are those with a GNI per capita of USD 13,846 or more in 2022 [13].

The Member States of the WHO are grouped into six regions: African Region (AFR), Region of the Americas (AMR), Eastern Mediterranean Region (EMR), European Region (EUR), South-East Asian Region (SEAR), and Western Pacific Region (WPR). These regions are organizational groupings, and while based on broad geography, they are not necessarily related to the geographical areas.

### 2.3. Data Analysis

We first described the countries’ incomes using the World Bank classification. We described the countries per WHO region and compared their 2024 incomes. The distribution of countries in each WHO region according to their income levels was analyzed using the chi-squared test. Differences at the 5% level (*p* < 0.05) were regarded as significant. We then described the TB CNR per countries’ income at the global level and in the six WHO regions from 1995 to 2022. The list of these countries is provided. All analyses were performed using Stata, version 18.1 ^®^ (Stat-Corp LP, College Station, TX, USA).

### 2.4. Ethics

Approval from an institutional review board was not required since this study did not involve human subjects.

## 3. Results

### 3.1. Description of Countries by Income, WHO Region, and TB Epidemiology

Of the 208 countries included in this study, the number and proportion by income stratus and by WHO region are shown in Table 1.

The distribution of countries in each of the WHO regions according to their income showed a number of significant differences (*p* < 0.001) (Table 2).

- In the African Region, 20 (42.6%) countries were categorized as low-income, 20 (42.6%) as lower-middle-income, 6 (12.7%) as upper-middle-income, and 1 (2.1%) as high-income.
- In the Region of the Americas (AMR), the European Region (EUR), and the Western Pacific Region (WPR), no countries were categorized as low-income. In these regions, high-income countries were predominant: 45.2% in AMR, 64.8% in EUR, and 43.7% in WPR.
- In the Eastern Mediterranean Region (EMR), 5 (22.7%) countries were categorized as low-income, 8 (36.4%) as lower-middle-income, 3 (13.6%) as upper-middle-income, and 6 (27.3%) as high-income.
- In the South-East Asian Region (SEAR), no countries were categorized as high-income. One (9.1%), 7 (63.6%), and 3 (27.3%) countries were categorized, respectively, as low-income, lower-middle-income, and upper-middle-income.

The detail list of the countries by income and by WHO region is shown in Table 3. These data are also shown in the Supplementary Materials.

### 3.2. Trend of the Mean TB CNR Rate from 1995 to 2022

The trends of the mean of the TB CNR by income category at the global level and by WHO region are described below. CNR data are also shown in the Supplementary Materials.

#### 3.2.1. TB CNRs from 1995 to 2022 by Income at Global Level

**Figure 1:**
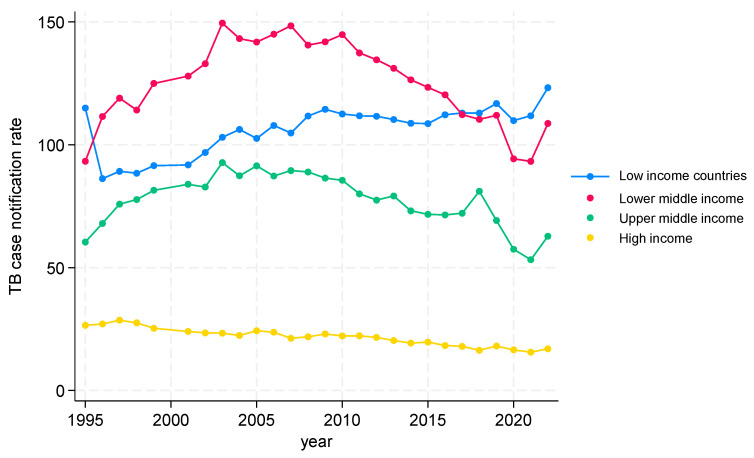
TB case notification at global level by incomes, 1995 to 2022.

TB CNRs at the global level are shown in Figure 1. In low-income countries, after an initial decrease, the TB CNR gradually increased, with a slight dip, followed by a rise from 2020 onwards. In lower- and upper-middle-income countries, the trends followed the same pattern—a gradual increase up to 2005, followed by a plateau for five years and then a decline with a marked dip, followed by a rise from 2020 onwards. In high-income countries, there was a gradual decline in the TB CNR over the time period. In general, over the whole study period, the low-income countries had TB CNRs between 100 and 120, while the lower-middle-income countries had higher rates between 120 and 150. Upper-middle-income countries had TB CNRs between 50 and 100, while high-income countries had TB CNRs at about 25 or lower.

#### 3.2.2. TB CNRs from 2000 to 2022 in WHO Regions by Income

##### TB CNRs from 1995 to 2022 in WHO African Region

**Figure 2:**
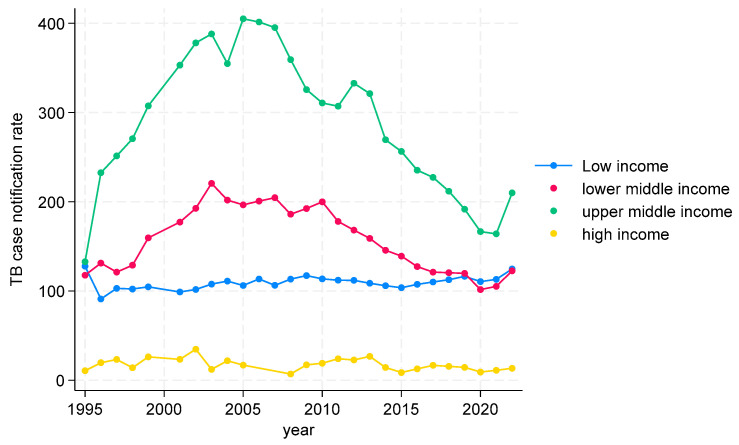
TB CNRs in WHO African Region by incomes, 1995 to 2022.

TB CNRs in the WHO African region are shown in Figure 2. In low-income countries, after an initial decrease, the TB CNR gradually increased, with a slight dip, followed by a rise from 2020 onwards, essentially following the trend shown at the global level in the previous figure. In the lower- and upper-middle-income countries, the trends followed the same pattern—a marked increase up to 2005, followed by a significant decrease with a further dip, followed by a rise from 2020 onwards. The TB CNR was higher in the upper-middle-income countries (between 200 and 400) compared with the lower-middle-income countries (between 100 and 200) during the whole period. In high-income countries, the TBCNR remained fairly stable at less than 20 during the study period.

##### TB CNRs from 1995 to 2022 in the WHO Region of the Americas (AMR)

**Figure 3:**
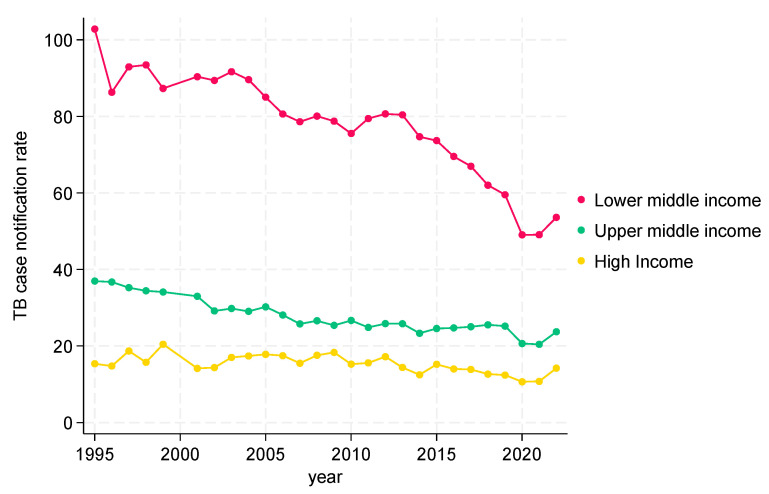
TB CNRs in WHO Region of the Americas by incomes, 1995 to 2022.

TB CNRs in the WHO Americas region are shown in Figure 3. There were no low-income countries. For the lower-middle-income, upper-middle-income, and high-income countries, there was an overall decline in TB CNRs, most marked in the lower-middle-income countries (a decline from 100 to 50), followed by the upper-middle-income countries (a decline from 40 to 20), and least marked in the high-income countries (below 20).

##### TB CNRs from 1995 to 2022 in WHO Eastern Mediterranean Region (EMR)

**Figure 4:**
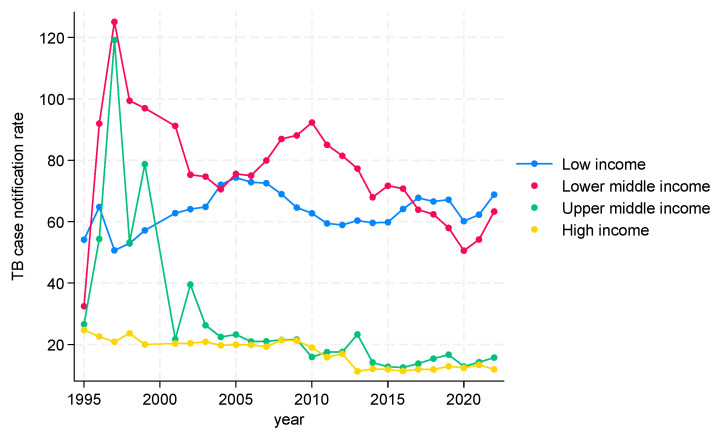
TB CNRs in WHO Eastern Mediterranean Region by incomes, 1995 to 2022.

TB CNRs in the WHO Eastern Mediterranean region are shown in Figure 4. There were some substantial variations over the 27-year period. In low-income countries, the trend was that of a gradual increase (rates starting below 60 and reaching about 70). In lower-middle-income countries, there was large spike between 1995 and 1997, followed by a decline, another spike, and then a decline (the rates being generally higher than in low-income countries at between 70 and 90). In upper-middle-income countries, it was the same general pattern, with a large initial spike, followed by a rapid decline and then a more gradual decline (rates remaining below 40 from 2002 onwards). In high-income countries, TB CNRs were relatively stable between 1995 and 2010, followed by a gradual decline with TB CNRs being 20 or below.

##### TB CNRs from 1995 to 2022 in WHO European Region (EUR)

**Figure 5:**
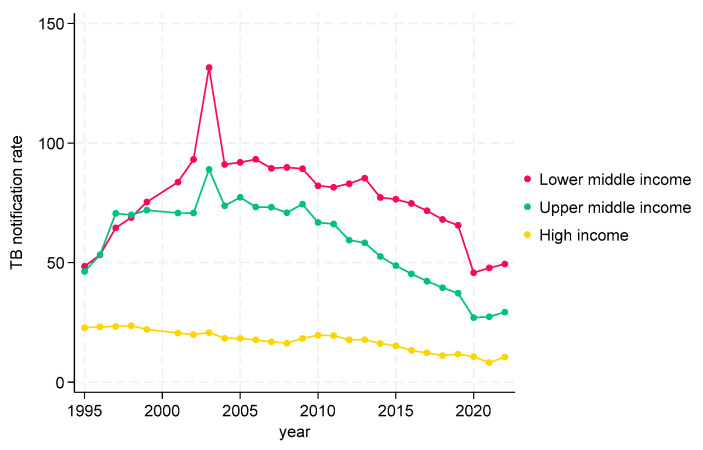
TB CNRs in WHO European Region by incomes, 1995 to 2022.

TB CNRs in the WHO European region are shown in Figure 5. There were no low-income countries. The lower- and upper-middle-income countries followed the same general pattern, with an increase in TB CNRs up to 2005, with a large spike in 2003 and 2004, followed by a gradual decline. In high-income countries, there was a slow gradual decline. Apart from the high spikes in 2003, TB CNRs in upper- and lower-middle-income countries declined from about 90 to below 50 over the study period.

##### TB CNRs from 1995 to 2022 in WHO South-East Asian Region (SEAR)

**Figure 6:**
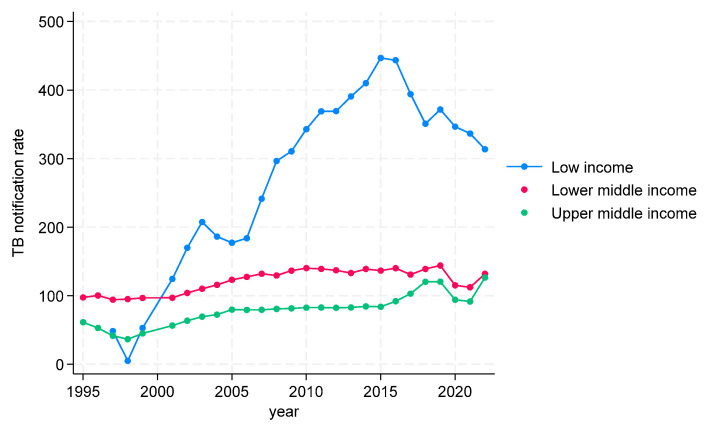
TB CNRs in WHO South-East Asian Region by incomes, 1995 to 2022.

TB CNRs in the WHO South-East Asian region are shown in Figure 6. In low-income countries, the TB CNRs initially dipped and then showed a striking increase up to 2015, at which point the CNRs declined, the TB CNRs being below 10 in 1998 and peaking at above 400 in 2015. Both upper- and lower-middle-income countries showed a gradual increase in TB CNRs over the study period, with lower-middle-income countries being at 100 to 120 and upper-middle-income countries being generally below 100 throughout. No high-income countries were represented.

##### TB CNRs from 1995 to 2022 in WHO Western Pacific Region (WPR)

**Figure 7:**
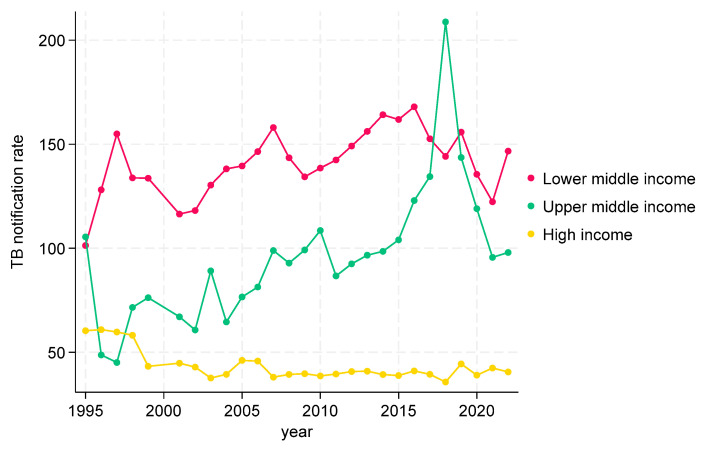
TB CNRs in WHO Western Pacific Region (WPR) by incomes, 1995 to 2022.

TB CNRs in the WHO Western Pacific region are shown in Figure 7. There were no low-income countries. In lower-middle-income countries, the TB CNRs fluctuated over the study period, but showed an overall increase followed by a decrease (rates varying from 100 to 150). Upper-middle-income countries also showed fluctuations, with an increase in CNRs with a dramatic spike between 2017 and 2018, followed by a dramatic decrease (rates varying from 70 to 100 apart from the large spike, which reached over 200). In high-income countries, there was an initial decrease, followed by generally stable TB CNRs with rates below 50 from 1998 onwards.

## 4. Discussion

### 4.1. Overview of Key Findings

This study provides a comprehensive analysis of TB CNRs from 1995 to 2022, categorized by income levels and across WHO regions. The analysis reveals significant disparities in TB CNRs, which are closely linked to income status and regional characteristics.

### 4.2. Income-Based Disparities in TB Notification Rates

One of the most striking findings was the stark contrast in TB CNRs between countries of different income levels. Low-income, lower-middle-income, and upper-middle-income countries consistently reported substantially higher TB CNRs compared to high-income countries. This disparity underscores the persistent and profound impact of socioeconomic factors on TB incidence and control [16,17].

In low-income countries, the high TB CNRs can be attributed to a combination of factors, including inadequate health care infrastructure, limited access to diagnostic and treatment services, and a higher prevalence of risk factors such as malnutrition, overcrowding, and co-infections like HIV. These challenges are often exacerbated by limited resources and political instability, making it difficult to implement and sustain effective TB control programs.

Lower-middle-income and upper-middle-income countries, while generally better resourced than low-income countries, still face significant barriers to reducing TB incidence. These countries often struggle with gaps in health care access, varying levels of health care quality, and challenges in managing TB in urban slums and rural areas where health care infrastructure is weak. Additionally, the rising burden of non-communicable diseases like diabetes mellitus, which is a known risk factor for TB, further complicates TB control efforts in these countries.

In contrast, high-income countries have significantly lower TB CNRs, which can be largely attributed to stronger health care systems, better access to quality health care, and more effective public health interventions. These countries have benefited from decades of investment in health care infrastructure, comprehensive TB control programs, and robust social safety nets that reduce the risk factors associated with TB transmission and progression.

### 4.3. Regional Trends and Implications

The regional analysis revealed significant variations in TB CNR trends, influenced by both income levels and regional factors.

In the African Region, the high TB CNRs in low- and lower-middle-income countries, ranging from 100 to 400, highlights the ongoing TB burden despite efforts to scale up TB control programs. The stability of TB CNRs in high-income countries in this region at levels below 20 suggests effective TB management is possible in those settings. However, in low- and middle-income countries, TB trends have almost certainly been exacerbated by high rates of HIV co-infection, malnutrition, and other socioeconomic challenges that increase the risk of TB transmission and progression [11].

The Region of the Americas (AMR) shows a clear income gradient, with no low-income countries and a marked decline in TB CNRs across income levels. The reduction in TB CNRs in lower-middle-income countries from 100 to 50 is particularly encouraging and reflects successful TB interventions. However, the persistent, albeit lower, rates in upper-middle and high-income countries suggest the need for continued vigilance, particularly in addressing risk factors such as diabetes and smoking, which are prevalent in these regions [11].

In the Eastern Mediterranean Region (EMR), the variability in TB CNRs, especially the large spikes followed by declines, suggests that TB trends are heavily influenced by factors such as conflict, migration, and health system disruptions. Additionally, high levels of poverty and malnutrition and increasing rates of diabetes in some parts of the region likely contribute to the observed trends, underscoring the need for integrated approaches to address these underlying risk factors alongside TB-specific interventions.

The European Region (EUR) and the Western Pacific Region (WPR) showed a general decline in TB CNRs across income levels, with the exception of some spikes in middle-income countries, particularly in 2003 and 2018 respectively. These spikes may reflect epidemiological transitions or the effects of policy changes and should be further investigated. The increasing prevalence of diabetes and aging populations in some countries within these regions could also contribute to the observed trends, requiring targeted interventions to mitigate these risk factors.

The South-East Asian Region (SEAR) presents a unique challenge, with a striking increase in TB CNRs in low-income countries, peaking at above 400 in 2015. This region’s trends indicate significant ongoing transmission, likely driven by high rates of malnutrition, poverty, and overcrowding, which create an environment conducive to TB spread. These findings highlight the need for intensified TB control efforts, particularly in lower-middle-income countries where rates remain high, and where addressing social determinants of health is critical to reducing the TB burden. In SEAR, compared to the African region, low-income countries presented higher TB CNRs than lower-middle-income countries and upper-middle-income countries. The disparities may be attributed to variations in health care system efficiency, disease surveillance capacities, and socioeconomic factors specific to each region. For instance, in the African Region, lower-middle-income countries and upper-middle-income countries may have higher CNRs due to better diagnostic capacity and reporting systems, while in SEAR, the association with poverty and health care access likely plays a larger role.

### 4.4. Impact of the COVID-19 Pandemic

Across all regions, the impact of the COVID-19 pandemic on TB CNRs was evident, with dips in 2020 followed by subsequent rises. This pattern underscores the vulnerability of TB control programs to global health emergencies and highlights the importance of building resilient health systems that can maintain essential services during crises [11,18].

### 4.5. Historical Context of TB in High-Income Countries

It is important to recognize that the current low TB CNRs in high-income countries is the result of a long historical decline in TB incidence and mortality. In the early 1900s, TB was a major public health issue in these countries, with high case notification rates, high morbidity, and high mortality [19]. The decrease in TB cases in high-income countries began well before the introduction of the first antibiotics and the widespread use of TB control programs. This decline is largely attributed to improving socioeconomic conditions, such as better nutrition, housing, and overall living standards, although natural selection may also have played a role [19]. Additionally, these countries developed effective surveillance systems that allowed them to monitor the TB epidemic closely and adapt their public health strategies accordingly. High-income countries maintained these low TB CNR levels throughout the study period by delivering adequate TB services and ensuring universal access to health care, either through state-funded systems or insurance-based models. This combination of historical socioeconomic improvements and ongoing health care access has been crucial in sustaining low TB rates.

### 4.6. Implications of Our Results for TB Control Strategies

TB is intricately linked to poverty and other socioeconomic determinants, as described in the published literature [20,21,22,23,24,25]. The findings of this study have critical implications for TB control strategies. The persistent high TB CNRs in low-income countries suggests that current strategies are insufficient and need to be bolstered with greater resources and tailored interventions that address the specific challenges of these regions. High-income countries have demonstrated the effectiveness of combining improved socioeconomic conditions with strong health care systems to reduce TB CNR. Low- and middle-income countries need to adopt a similar approach, focusing on providing universal health coverage, delivering high-quality TB care and prevention services, and addressing social determinants of TB such as HIV, diabetes, malnutrition, smoking, and alcohol use. These strategies can be effectively summarized into ten key areas, which are shown in Table 4. This table provides a clear and concise overview of the critical components necessary to strengthen TB control efforts globally, particularly in low- and middle-income countries. Adopting a Holistic Approach would be highly beneficial in tackling TB, particularly in low- and middle-income countries. This would involve not only strengthening health care systems to ensure universal health coverage and high-quality TB care, but also addressing broader social determinants of health. These include, for example, tackling poverty, improving nutrition, reducing smoking and alcohol abuse, managing co-morbidities like HIV and diabetes, and enhancing public health education. By focusing on these interconnected factors, countries can create a sustainable framework for reducing TB incidence and improving overall public health outcomes.

### 4.7. Limitations and Future Research

This study, while comprehensive, is limited by its reliance on reported TB CNRs, which may not fully capture the true burden of TB, particularly in regions with weaker surveillance systems. Additionally, factors such as migration, conflict, and socioeconomic changes, which can significantly impact TB trends, were not fully explored. Future research should aim to incorporate more detailed data on these factors and investigate the long-term impacts of the COVID-19 pandemic on TB control efforts. Another limitation of this study is the use of 2024 income classifications from the World Bank to categorize countries over the entire 27-year period. This approach provides a consistent framework for analysis, but does not account for transitions in income classifications that may have occurred during this timeframe. For instance, some countries may have moved from low- to middle-income status or vice versa due to economic growth, political changes, or other factors. These transitions could impact the interpretation of trends in TB case notification rates, as the socioeconomic conditions influencing the TB burden may have shifted over time. Future studies could explore the effects of dynamic income classification on TB trends to provide a more nuanced understanding of these relationships.

## 5. Conclusions

The findings from this study reinforce the well-established understanding that TB is a disease of poverty. The relationship between income and TB incidence is clear: As income levels decrease, TB CNRs rise. This reflects the broader social determinants of health, where poverty exacerbates vulnerability to TB by limiting access to health care, increasing exposure to risk factors, and reducing the ability to complete treatment. This income-related disparity in TB CNRs also highlights the urgent need for targeted interventions in low-income, lower-middle-income, and upper-middle-income countries. Addressing the root causes of TB, including poverty and inequality, is essential for reducing the global burden of the disease. This requires a multifaceted approach that not only improves access to health care, but also addresses the social determinants of health that drive TB transmission and progression.

## Figures and Tables

**Table 1:** Description of the income and WHO region of the countries included in the study.

Characteristics	*n* (%)
Income	
Low income	26 (12.5)
Lower-middle income	54 (26.0)
Upper-middle income	53 (25.5)
High income	75 (36.1)
WHO regions	
African Region (AFR)	47 (22.6)
Region of the Americas (AMR)	42 (20.2)
Eastern Mediterranean Region (EMR)	22 (10.6)
European Region (EUR)	54 (26.0)
South-East Asian Region (SEAR)	11 (5.3)
Western Pacific Region (WPR)	32 (15.4)

**Table 2:** Comparison of country income by WHO region.

	Low Income	Lower Middle Income	Upper Middle Income	High Income	*p* Value
WHO Region					<0.001
African Region (AFR) (*n* = 47)	20 (42.6)	20 (42.6)	6 (12.7)	1 (2.1)	
Region of the Americas (AMR) (*n* = 42)	0 (0.0)	4 (9.5)	19 (45.2)	19 (45.2)	
Eastern Mediterranean Region (EMR) (*n* = 22)	5 (22.7)	8 (36.4)	3 (13.6)	6 (27.3)	
European Region (EUR) ( *n* = 54)	0 (0.0)	4 (7.4)	15 (27.8)	35 (64.8)	
South-East Asian Region (SEAR) (*n* = 11)	1 (9.1)	7 (63.6)	3 (27.3)	0 (0.0)	
Western Pacific Region (WPR) (*n* = 32)	0 (0.0)	11 (34.4)	7 (21.9)	14 (43.7)	
Total	26	54	53	75	

**Table 3:** List of countries in each WHO region by income group.

WHO Region	Income Categories	Countries
African Region (AFR)	Low income (*n* = 20)	Mali, Togo, Democratic Republic of the Congo, South Sudan, Chad, Burkina Faso, Gambia, Liberia, Sierra Leone, Mozambique, Guinea-Bissau, Central African Republic, Malawi, Madagascar, Uganda, Eritrea, Burundi, Rwanda, Ethiopia, Niger
	Lower-middle income (*n* = 20)	Algeria, Senegal, Nigeria, Cabo Verde, United Republic of Tanzania, Zambia, Comoros, Lesotho, Zimbabwe, Kenya, Côte d’Ivoire, Mauritania, Guinea, Congo, Cameroon, Angola, Sao Tome and Principe, Ghana, Eswatini, Benin
	Upper-middle income (*n* = 6)	South Africa, Namibia, Botswana, Equatorial Guinea, Gabon, Mauritius
	High income (*n* = 1)	Seychelles
Region of the Americas (AMR)	Low income (*n* = 0)	-
	Lower-middle income (*n* = 4)	Honduras, Nicaragua, Bolivia (Plurinational State of), Haiti
	Upper-middle income (*n* = 19)	Paraguay, Peru, Colombia, Dominican Republic, Guatemala, Saint Vincent and the Grenadines, Grenada, Brazil, Jamaica, Belize, Ecuador, Dominica, El Salvador, Argentina, Mexico, Saint Lucia, Suriname, Costa Rica, Cuba
	High income (*n* = 19)	Guyana, Bahamas, Puerto Rico, British Virgin Islands, Bermuda, Trinidad and Tobago, Cayman Islands, United States of America, Saint Kitts and Nevis, Antigua and Barbuda, Panama, Canada, Aruba, Barbados, Uruguay, Chile, Turks and Caicos Islands, Sint Maarten (Dutch part), Curaçao
Eastern Mediterranean Region (EMR)	Low income (*n* = 5)	Afghanistan, Yemen, Sudan, Somalia, Syrian Arab Republic
	Lower-middle income (*n* = 8)	Morocco, Djibouti, Egypt, Tunisia, Jordan, Lebanon, Iran (Islamic Republic of), Pakistan
	Upper-middle income (*n* = 3)	Occupied Palestinian territory, including east Jerusalem, Iraq, Libya
	High income (*n* = 6)	United Arab Emirates, Bahrain, Saudi Arabia, Kuwait, Qatar, Oman
European Region (EUR)	Low income (*n* = 0)	-
	Lower-middle income (*n* = 4)	Ukraine, Uzbekistan, Tajikistan, Kyrgyzstan
	Upper-middle income (*n* = 15)	Republic of Moldova, North Macedonia, Albania, Armenia, Turkmenistan, Kazakhstan, Azerbaijan, Georgia, Tăźrkiye, Russian Federation, Belarus, Bulgaria, Bosnia and Herzegovina, Montenegro, Serbia
	High income (*n* = 35)	Norway, Belgium, Germany, Romania, San Marino, Luxembourg, Slovenia, Sweden, Latvia, Lithuania, Malta, Iceland, Monaco, Italy, Croatia, Israel, Portugal, Denmark, Slovakia, Greenland, Finland, Greece, Austria, Andorra, Spain, Switzerland, Estonia, Ireland, Netherlands (Kingdom of the), Poland, United Kingdom of Great Britain and Northern Ireland, Czechia, Cyprus, Hungary, France
South-East Asian Region (SEAR)	Low income (*n* = 1)	Democratic People’s Republic of Korea
	Lower-middle income (*n* = 7)	Bhutan, Nepal, Bangladesh, India, Myanmar, Sri Lanka, Timor-Leste
	Upper-middle income (*n* = 3)	Maldives, Indonesia, Thailand
	High income (*n* = 0)	-
Western Pacific Region (WPR)	Low income (*n* = 0)	-
	Lower-middle income (*n* = 11)	Samoa, Solomon Islands, Lao People’s Democratic Republic, Mongolia, Kiribati, Viet Nam, Philippines, Papua New Guinea, Vanuatu, Micronesia (Federated States of), Cambodia
	Upper-middle income (*n* = 7)	Malaysia, Palau, Tuvalu, Marshall Islands, Fiji, Tonga, China
	High income (*n* = 14)	Singapore, American Samoa, Japan, China Macao SAR, China Hong Kong SAR, Nauru, New Caledonia, Guam, Northern Mariana Islands, Australia, French Polynesia, New Zealand, Brunei Darussalam, Republic of Korea

**Table 4:** Key areas for strengthening TB control strategies.

Key Area	Description
1. Strengthening Health Systems	Universal Health Coverage: Ensure all individuals have access to quality health services without financial hardship, including diagnostics, treatment, and preventive services.
	Health care Infrastructure: Invest in robust health care infrastructure, especially in low- and middle-income countries, to improve TB detection, treatment, and follow-up.
2. Enhancing TB Detection and Diagnosis	Advanced Diagnostics: Deploy advanced and rapid diagnostic tools, such as molecular tests (e.g., GeneXpert), to quickly and accurately identify TB cases. While GeneXpert has been a significant advancement in TB diagnostics, its utility in peripheral and resource-limited settings remains constrained due to its cost, infrastructure requirements, and operational challenges. These limitations highlight the urgent need for diagnostic tools that are affordable, portable, and capable of functioning effectively in decentralized settings with minimal resources. Future strategies to improve TB detection should prioritize innovations that address these gaps, ensuring accessibility and scalability across diverse health care systems.
	Screening Programs: Implement comprehensive screening programs targeting high-risk populations, including people living with HIV, health care workers, and those in close contact with TB patients.
3. Ensuring Effective Treatment and Care	Adherence to Treatment: Improve patient adherence to TB treatment regimens through education, support programs, and adherence-monitoring technologies.
	Drug-Resistant TB: Develop and distribute effective treatment regimens for multidrug-resistant TB (MDR-TB) and extensively drug-resistant TB (XDR-TB).
4. Addressing Social Determinants of Health	Poverty Reduction: Tackle poverty, malnutrition, and poor living conditions, which are significant risk factors for TB.
	Education and Awareness: Increase public awareness about TB prevention, symptoms, and the importance of completing treatment through educational campaigns.
5. Expanding Preventive Measures	Vaccination: Develop and distribute an effective TB vaccine. The current BCG vaccine has limited effectiveness in adults, so new vaccines are crucial.
	Preventive Therapy: Provide preventive therapy for individuals at high risk of developing TB, such as people living with HIV as well as household contacts of infectious pulmonary TB patients and those with latent TB infection.
6. Strengthening Global Collaboration and Funding	International Partnerships: Foster collaboration among countries, international organizations (e.g., WHO, Global Fund), NGOs, and research institutions to share knowledge, resources, and best practices.
	Sustainable Funding: Secure long-term funding for TB control programs to ensure consistent and comprehensive efforts.
7. Implementing Robust Surveillance and Research	Surveillance Systems: Strengthen TB surveillance systems to monitor trends, identify outbreaks, and measure the impact of interventions.
	Research and Innovation: Invest in research to develop new diagnostics, treatments, and vaccines and in operational research to understand how best to deploy new tools. Innovation and subsequent deployment are essential to address emerging challenges like drug-resistant TB.
8. Integrating TB Services with Other Health Programs	TB and Comorbidities Co-management: e.g., Integrate TB and HIV services to ensure that people living with HIV receive regular TB screening and prompt treatment if necessary. The same approach should be used for the other key risk factors (undernutrition, tobacco use, diabetes, and alcohol use).
	Primary Health Care: Incorporate TB services into primary health care to increase accessibility and reduce stigma.
9. Community Engagement and Support	Community Health Workers: Train and deploy community health workers to support TB detection, treatment adherence, and patient education.
	Empower Communities: Engage communities in TB control efforts to foster local ownership and culturally appropriate interventions.
10. Policy and Advocacy	National TB Programs: Strengthen national TB programs with clear policies, strategies, and guidelines aligned with global standards.
	Advocacy: Advocate for political commitment and leadership at all levels to prioritize TB control and allocate necessary resources.

## Data Availability

The data that support the findings of the study are available for public on the website of WHO and The World Bank.

## Supplementary Materials

The following supporting information can be downloaded at https://www.mdpi.com/article/10.3390/tropicalmed9120294/s1: Table S1: Countries CNR Income and WHO region.
